# Experts consensus on management of tooth luxation and avulsion

**DOI:** 10.1038/s41368-024-00321-z

**Published:** 2024-09-26

**Authors:** Ruijie Huang, Chenchen Zhou, Ling Zhan, Yuan Liu, Xian Liu, Qin Du, Jun Wang, Wei Zhao, Guangtai Song, Li-an Wu, Beizhan Jiang, Yanhong Li, Hongmei Zhang, Jing Zou

**Affiliations:** 1 State Key Laboratory of Oral Diseases & National Center for Stomatology & National Clinical Research Center for Oral Diseases & Department of Pediatric Dentistry, West China Hospital of Stomatology, Sichuan University, Chengdu, China; 2 Department of Orofacial Sciences, School of Dentistry, University of California, San Francisco, San Francisco, CA USA; 3 Division of Pediatric Dentistry, Preventative & Restorative Sciences, School of Dental Medicine, University of Pennsylvania, Philadelphia, PA USA; 4 State Key Laboratory of Oral Diseases, National Center for Stomatology, National Clinical Research Center for Oral Diseases, Department of Emergency, West China Hospital of Stomatology, Sichuan University, Chengdu, China; 5 Department of Stomatology, Sichuan Provincial People’s Hospital, University of Electronic Science and Technology of China, Chengdu, China; 6 Shanghai Ninth People’s Hospital, Shanghai Jiao Tong University School of Medicine, College of Stomatology, National Center for Stomatology, National Clinical Research Center for Oral Diseases, Shanghai Key Laboratory of Stomatology, Shanghai Research Institute of Stomatology, Shanghai, China; 7 Department of Pediatric Dentistry, Guanghua School of Stomatology, Hospital of Stomatology, Sun Yat-Sen University, Guangzhou, China; 8 Department of Pediatric Dentistry, School and Hospital of Stomatology, Wuhan University, Wuhan, China; 9 Department of Pediatric Dentistry, School of Stomatology, State Key Laboratory of Military Stomatology, National Clinical Research Center for Oral Diseases, Shanxi Key Laboratory of Military Stomatology, Fourth Military Medical University, Xi’an, China; 10 Department of Pediatric Dentistry, Stomatological Hospital and Dental School of Tongji University, Shanghai, China; 11 Department of Pediatric and Preventive Dentistry, Kunming Medical University School and Hospital of Stomatology, Kunming, China; 12 Department of Pediatric Dentistry, The Affiliated Stomatological Hospital of Chongqing Medical University, Chongqing Key Laboratory of Oral Diseases and Biomedical Sciences, Chongqing, China

**Keywords:** Dental diseases, Trauma

## Abstract

Traumatic dental injuries (TDIs) of teeth occur frequently in children and adolescents. TDIs that impact the periodontal tissues and alveolar tissue can be classified into concussion, subluxation, extrusive luxation, intrusive luxation, lateral luxation, and avulsion. In these TDIs, management of injured soft tissue, mainly periodontal ligament, and dental pulp, is crucial in maintaining the function and longevity of the injured teeth. Factors that need to be considered for management in laxation injuries include the maturation stage of the traumatic teeth, mobility, direction of displacement, distance of displacement, and whether there are alveolar fractures. In avulsion, the maturation stage of the permanent tooth, the out-socket time, storage media/condition of the avulsed tooth, and management of the PDL should also be considered. Especially, in this review, we have subdivided the immature tooth into the adolescent tooth (Nolla stage 9) and the very young tooth (Nolla stage 8 and below). This consensus paper aimed to discuss the impacts of those factors on the trauma management and prognosis of TDI to provide a streamlined guide for clinicians from clinical evaluation, diagnostic process, management plan decision, follow-up, and orthodontic treatment for tooth luxation and avulsion injuries.

## Introduction

Traumatic dental injuries (TDIs) most commonly occur in children and young adults. Twenty-five percent of all school children experience dental trauma^1^ and 33% of adults have experienced trauma to the permanent dentition.^2^ Overall, about one billion people have experienced TDIs.^3^ Luxation injuries are the most common TDIs in primary dentition, whereas crown fractures are more commonly reported for permanent teeth.^2^ Males suffer more TDIs than females.^4^ The most widely used dental trauma classification system was developed by Dr. Jens Ove Andreasen in the 1970s.^5^ Dr. Andreasen has classified TDIs into four types, injuries to the hard dental tissues and pulp, injuries to the periodontal tissues, injuries to the supporting bone, and injuries to the gingiva or oral mucosa. Among the four types, the injuries to the periodontal tissues are usually more complicated and require longer follow-ups. The injuries to the periodontal tissues are also known as tooth displacement injuries, including five different types of tooth luxation and tooth avulsion. The five different types of tooth luxation are concussion, subluxation, extrusive luxation, intrusive luxation, and lateral luxation.^6,7^ In Xi’an, China, the prevalence of concussion, subluxation, extrusive luxation, intrusive luxation, and lateral luxation is 27.4%, 21.5%, 16.3%, 11.1%, 5.9%, and 17.8%, respectively, in tooth luxation and avulsion category.^8^ The prevalence of luxation in 1–6, 7–12, and >13-year-olds are 38.5%, 57.0%, and 4.4%, respectively.^8^ In Hongkong, China, the prevalence of tooth concussion, subluxation, extrusive luxation, intrusive luxation, and lateral luxation is 35.2%, 50.1%, 5.9%, 2.5%, 1.1%, and 5.1%, respectively, in primary school children tooth luxation and avulsion.^9^ If tooth luxation and avulsion have not been managed properly, this would lead to poor prognosis and generate lifelong impact on the patient.^2^ The understanding of pathologic changes and proper management would result in better outcomes. Therefore, the goal of this consensus is to discuss the soft and hard tissue changes in the occurrence and recovery of tooth luxation and avulsion and to provide suggestions for trauma management based on those changes. The clinical and radiographic observations, dental treatment, emergency treatment and post-trauma care, possible complications and countermeasures, and the orthodontic considerations for traumatic teeth will be discussed.

## Pathologic changes and related symptoms

### The series of pathological changes in soft tissue ischemia

The tissue cells’ growth requires a proper physiologic environment, with a blood supply that brings nutrients and oxygen to the tissue. However, if the blood supply is compromised, known as ischemia, this will lead to damage of the cells and dysfunction of the tissues. In ischemia, the removal of metabolic wastes would also be compromised, leading to accumulated toxic metabolites in the tissue.^10^ In prolonged ischemia, due to the anaerobic metabolism and lactate accumulation, the ATP levels and intracellular pH would decrease. As a result, the ATPase-dependent ion transport system becomes dysfunctional, leading to increased intracellular and mitochondrial calcium levels, cell swelling and rupture, and cell death through necrosis, apoptosis, necroptosis, and autophagy mechanisms.^11^ In an extreme situation, with the presence of mono- or multiply-species bacterial infection, the necrotizing soft tissue infection may occur, leading to severe tissue inflammation and necrosis.^12^

### Pulp tissue changes in luxation and avulsion

The dental pulp is the innermost tissue of the tooth. It contains blood vessels, nerves, connective tissues, and specialized cells, and provides nutrients for the tooth.^13^ Unlike the periodontal ligament, there is only one main blood vessel coming from the apical foramen of the root apex. The blood supply brings oxygen and nutrients to the pulp tissue.^14^

#### Sub-classification of immature tooth

The International Association for Dental Traumatology (IADT) guidelines have classified the tooth into immature tooth (apex open) and mature tooth (apex closed).^7^ The mature tooth refers to a tooth in Nolla tooth development stage 10, and the immature tooth refers to a tooth in Nolla stages 9 and below (Table 1). According to IADT guidelines, for an immature avulsed tooth, since the apex foramen is open, the proper treatment is to preserve the pulp and monitor the pulp condition in recalls.^7^ However, in clinic practice, we noticed that the outcome of pulp reservation is much worse in an immature tooth of Nolla stage 9 than an immature tooth of Nolla stage 8 or 7. Therefore, in the current review, we have subdivided the immature tooth into adolescent tooth (Nolla stage 9) and very young tooth (Nolla stage 8 and below). The adolescent tooth is like a froglet, which is in a transitional stage between a mature tooth and a very young tooth (Table 1).

**Table 1:** Stages of tooth develop

Nolla stages	IADT classification	West China classification
10: Full root length develops, apical foramen closed	Mature tooth	Mature tooth
9: Full root length develops, apex open	Immature tooth	Adolescent tooth
8: 2/3 Root length develops, apex open	Immature tooth	Very young tooth
7: 1/3 Root length develops, apex open	Immature tooth	Very young tooth

#### Factors affect the clinical outcome

Several factors, stage of tooth development, distance of dislocation, and mobility of the traumatic tooth, are associated with the outcome of tooth luxation or avulsion.

##### Stage of tooth development

There are three factors that affect tissue healing, tissue dehydration, blood supply, and sepsis.^14^ Tissue regains blood supply via angiogenesis, in which capillary grows and vascular bedforms.^15^ In a luxation tooth, the recovery of pulp blood supply partially depends on the diameter of the apical foramen. The tooth with a larger foramen has a larger contact area for revascularization, therefore, the chance of successfully regaining blood supply is higher in a tooth with a large foramen than in a tooth with a smaller foramen. That is, a very young tooth is more likely to regain blood supply than an adolescent tooth and a mature tooth.^16^

##### Distance of dislocation

There is limited elasticity of the blood vessel. If the dislocation distance has exceeded the elastic limit of the blood vessel, the blood vessel breaks. Therefore, in general, the shorter the tooth dislocates, the better the blood vessel recovers. A luxation tooth with less dislocation usually results in better outcomes.^17^

##### Mobility of the traumatic tooth

As stated above, there is limited elasticity of the blood vessel. If the force has exceeded the elastic limit of the blood vessel, the blood vessel breaks. The force is usually associated with tooth mobility. Therefore, in general, the less mobility the tooth is, the better the blood vessel recovers. A luxation tooth with less mobility usually results in better outcomes than one with more mobility.^18^

#### Indicators of pulp status

Blood flow, also known as hemoperfusion, is a direct indicator of pulp status. Laser Doppler flowmetry can be used to measure the blood flow.^19^ However, if laser Doppler flowmetry is not available, the tooth discoloration may reflect the blood supply and pulp status to a certain extent. If the tooth is pale, this indicates less blood flow rate, usually accompanied by low blood oxygen saturation.^20^ If the tooth is reddish or pinkish, this indicates the hemorrhage, as the blood is accumulated in the site with high carbon dioxide concentration, which is usually accompanied by edema.^21^ If the tooth is grayish, this usually indicates a necrotic pulp status. Since some dark discolored primary teeth can be preserved for a long time in some cases, it is still a dilemma if the endodontic treatment should be carried for a dark-colored tooth.^22^ But for the light discolored tooth, the color change of the tooth should be followed until there are some other evidence coming up to support the estimation of the pulp status. In some cases, the light-discolored tooth would recover by itself.^23^

Continuous root development is another direct indicator of live pulp status in immature teeth. This can be observed through radiographs in follow-up dental visits.

#### Dental pulp post-trauma healing

There are three types of dental pulp post-trauma healing, normal pulp, pulp necrosis, and pulp canal obliteration.

##### Normal pulp

This usually happens in mild luxation or very young teeth. The dental pulp maintains the normal function and the shape of the pulp canal is normal.

##### Pulp canal obliteration

This usually happens in moderate or severe luxations, such as extrusive luxation, intrusive location, and lateral luxation, with an open apex.^6,24^ When soft tissue continuously suffers ischemia or trauma, the odontoblastic secretory activity will lose neural control, resulting in accelerated dentin deposition and pulp canal obliteration.^25^

##### Pulp necrosis

This usually happens in severe luxation or avulsion, when infection presents.^24^

### Periodontal ligament changes in luxation and avulsion

#### PDL injury in luxation and avulsion

The periodontal ligament is a connective tissue in between of cementum and periodontal bone (alveolar process). The main components of the periodontal ligament are principal fibers, including alveolar crest fibers, horizontal fibers, oblique fibers, apical fibers, and inter-radicular fibers.^26^ The periodontal ligament provides the tooth attachment to the alveolar bone, and the width of the periodontal ligament is around 0.15–0.21 mm on radiographs.^27^ The periodontal ligament can disperse the occlusal force from the tooth crown to the alveolar bone, protecting the tooth from extra force load.^28^ Three major blood vessels nourish the periodontal ligament. They are perforating vessels, apical vessels, and gingival vessels, from which the periodontal ligament gets nutrients and removes waste away, in order to maintain the physiologic functions of the cells in the periodontal ligament.^10^

In tooth luxation, no matter with or without tooth dislocation, since the periodontal ligament attaches to its blood supply, laceration presences in some cases, and the overall recovery of the periodontal ligament is good.^6^

In tooth avulsion, since the periodontal ligament has been cut from its blood supply, the recovery of the periodontal ligament depends on how long the tooth has been kept dry outside the alveolar socket and whether it gets contaminated by the soil or not.^7^ Usually, if the tooth can be put back immediately, the status of the periodontal ligament is usually good. If the tooth has been kept dry for less than 60 min, the status of the periodontal ligament is fair, with some cells having already died. If the tooth has been kept dry for more than 60 min, the status of the periodontal ligament is poor, with the majority of cells having already died.^7^ Therefore, it is critical to put the tooth back into the alveolar socket as soon as possible. If on-site replantation is not available, the tooth should be kept in a physiologic media and sent to the dental clinic quickly. Milk, saline, and some commercial tooth preservation kits are usually used to preserve the avulsed tooth. One meta-analysis indicated that Hank’s balanced salt solution (HBSS), oral rehydration salts, propolis, and even rice water and cling film have been proven to be more effective in cell viability preservation than milk, yolk is similar compared to milk, but saline is less effective.^29^

#### PDL Treatment

It is still controversial whether the periodontal ligament should be removed or not from the root surface if the avulsed tooth has been kept dry for more than 60 min. If the periodontal ligament has been removed, the cementum would contact with the alveolar bone directly, in this case, ankylosis will easily happen. However, if the periodontal ligament has been preserved since some cells and tissue have already become necrotic, inflammation may happen, which can also lead to root resorption and tooth ankylosis. In 2012 IADT guidelines, the periodontal membrane on the tooth surface was suggested to be removed^30^ while in 2020 IADT guidelines, the membrane was suggested to be cleaned by agitation but not removed.^7^ In some studies, concentration growth factors (CGF) membrane and platelet-derived growth factors, which are derived from blood, have been used in periodontal ligament regeneration.^31^ The application of CGF in avulsed tooth treatment need more large-scale clinic research support before it is widely recommended.

#### PDL post-trauma healing

There are three types of PDL post-trauma healing, normal PDL, ankylosis, and external resorption.

##### Normal PDL healing

This usually happens in mild luxation or on-site tooth replantation of a very young tooth. This is the best PDL healing with normal functional PDL tissues.

##### Ankylosis

It is a condition that the periodontal ligament disappears totally or partially and the cementum fuses with the alveolar bone. The bone tissue replaces the periodontal ligament, leading to a fixation of the tooth to the bone.^32^ Since the craniofacial bones continuously develop until adulthood, if the ankylosis happens in children and adolescents, the traumatic tooth usually becomes “shorter” than the unaffected ones because it cannot erupt properly. The root is held back by the alveolar bone during craniofacial development, and the tooth cannot move to its ideal occlusal position. If the ankylosis happens in adulthood, although the root fuses to the bone, since there is no more craniofacial development, the traumatic tooth usually looks “the same height” as a normal companion tooth. But the high-metallic sound is present in the ankylosed tooth, no matter whether the trauma happens in childhood or in adulthood, since this indicates the fusion of root and alveolar bone. For the treatment of an ankylosed tooth, please refer to the “Follow-up, prognosis, and management of complications” section of this review.

##### External inflammatory resorption

The external inflammatory resorption is aggressive and destructive.^33^ If the microorganisms are present on the root surface, they generate toxins, which will recruit osteoclasts and inflammatory cells and result in bone resorption. Moreover, the lack of blood supply to the affected tooth would aggravate this process.^33^ Corticosteroid-antibiotics can be used intracanal to control the external resorption. Calcium hydroxide should not be firstly used because of its toxicity to the cells, but it can be used after the corticosteroid-antibiotics to stimulate root hard tissue deposit.^34^

## Clinical evaluation and diagnostic process

### History

#### Medical history

The medical history is used to acknowledge the patient’s past or current health status. It usually includes past and present diagnoses, medical care, treatments, and allergies.

#### Medication information

This is also known as drug therapy. The dentist should review this information and decide the medication that the patient would take. If the dentist is not certain about some special medications, he should refer to the patient’s physician for more information.

#### Family history

Family history may help to hint at some genetic or hereditary diseases. Since tooth luxation or avulsion are traumatic diseases, a protruded anterior tooth may be genetically acquired and contribute to the occurrence of TDIs.^8^

#### Treatment history

It usually includes chief complaints, history of illness, vital signs, physical examination, surgical history, medical allergies, immunization history, habits, etc.

### Clinical evaluation

#### Percussion

In dentistry, a percussion test is usually used to assess apical inflammation. However, for a traumatic tooth, a percussion test is usually used to assess the periodontal membrane status. If percussion generates tenderness, it indicates a traumatic status of the periodontal membrane. While if percussion does not generate any discomfort, it indicates a healthy status. This is useful especially when the percussion is tender in the first visit but recovers in follow-ups, which indicates good healing of the periodontal ligament. For intrusive and lateral luxation, it gives a high metallic (ankylotic) sound, because the root is stuck in the alveolar bone and becomes immobile.

#### Gingival bleeding

Gingival bleeding indicates periodontal ligament laceration.

#### Mobility

Miller’s tooth mobility grading system is adopted in this review.^35^ Briefly, 0 = no mobility, 1 = greater than normal (physiological), 2 = <1 mm in buccolingual direction, and 3 = >1 mm in buccolingual direction and depressible (apicocoronally movable). Teeth with more mobility have a higher risk of pulp damage, especially in a mature tooth with a closed apex.^24^

#### Tooth dislocation

The direction and distance should be recorded. The direction of tooth dislocation partially determines tooth diagnosis. The distance indicates the severity of tooth dislocation. In some treatments, such as intrusive luxation, the distance partially determines the treatment plan.^6^

#### Dental pulp testing

Dental pulp testing is discussed in a separate subsection “The application of dental pulp testing”.

### Radiographic evaluation

For radiographic evaluation, a periapical radiograph is the first choice.^36^ However, cone beam computed tomography (CBCT) radiograph can also be used to estimate the three-dimensional direct of tooth displacement and the alveolar socket wall fracture or compression and assess whether the dental displacement is accompanied by or results in jaw fracture or damage of surrounding tissue such as maxilla sinus, nasopalatine nerve, etc.^36^ For a very young child, the CBCT should be used with caution because of ionizing radiation. CBCT is usually not the first choice of radiographic evaluation in most cases.

#### Tooth dislocation

The direction and distance should be recorded.

#### Periodontal space

This is used to facilitate estimating the direction and severity of tooth dislocation and periodontal ligament damage.

#### Root fracture

Need to rule out root fracture.

### The application of dental pulp testing

The dental pulp testing has been widely used to estimate the status of dental pulp. Dental pulp testing can be classified into pulp sensibility test and pulp viability. The pulp sensibility test assesses the pulp’s sensory response, in other words, tests whether the pulp has a neural response to a stimulus or not. The typical pulp sensibility tests are electric pulp testing (EPT) and thermal testing. The pulp viability test assesses the pulp’s blood supply, in other words, tests whether the blood supply to the pulp has been compromised or not. The typical pulp viability tests are pulse oximetry and Laser Doppler flowmetry.^37^ The pulp viability test is considered superior to the pulp sensibility test because it measures the true health of the dental pulp. However, since there are still some practical issues with the pulp viability test, the pulp sensitivity test is the most frequently used pulp test in clinical practice.^37^

#### Types of dental pulp testing

##### Thermal testing

Pulp thermal tests are divided into hot testing and cold testing. The hot testing applies a hot stimulus, such as heated gutta percha, on the tooth surface and tests if the tooth can sense this hot or not. Like hot testing, cold testing applies a cold stimulus, such as ice, dry ice, ethyl chloride, dichlorodifluoromethane, etc., on the tooth and tests if the tooth can sense this cold or not.^37^ For toddlers and young children, thermal testing is not recommended for the first dental visit, because toddlers and young children are not able to correctly express their feelings immediately after the trauma, moreover, the pulp is usually in shock status at this visit and the result is not that reliable. Especially, avoiding hot testing on toddlers and young children since this would generate pain and even iatrogenic injury on them.

##### Electric pulp testing (EPT)

The EPT generates electrical stimuli, which cause ionic change across the neural membrane, and as the voltage increases the patient feels a “tingling” sensation.^37^

##### Pulse oximetry and laser Doppler flowmetry

Pulse oximetry is a noninvasive test for monitoring a tooth’s blood oxygen saturation,^37^ and laser Doppler flowmetry is used to assess pulp blood flow.^38^ Pulse oximetry provides higher diagnostic accuracy than EPT and thermal testing.^39^ However, due to insufficient reports, the diagnostic accuracy of laser Doppler flowmetry cannot be predicted at present.^40^

#### Dental pulp testing decision tree

**Fig. 1:**
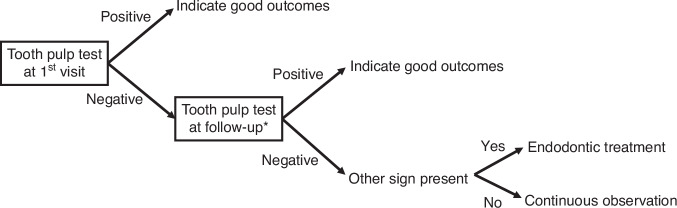
Dental pulp testing decision tree. *Please refer to Table 5 for detailed follow-up visit time points

For traumatic teeth, especially for an immature traumatic tooth with an open apex, the result of dental pulp testing is not accurate.^41^ An open apex tooth can give a false negative EPT. A traumatic tooth in shock condition, with a sudden drop in blood flow, with lead to negative results in pulse oximetry and laser Doppler flowmetry. Even if false results are generated, the dental pulp testing should be carried out at the first dental visit and follow-up. If the pulp test is positive at the first dental visit, this indicates that the pulp is in good condition and the pulp is likely could be preserved.^42^ However, if the pulp test is negative at the first dental visit, the dental pulp testing should be carried out in each follow-up, until the result turns positive or there are other signs to support the pulp necrosis, which needs pulp therapy.^6^ For easier decision-making in each dental visit, a dental pulp testing decision tree has been generated (Fig. 1).

### Summary of clinical and radiographic observations and diagnosis

#### Clinical findings

The main clinical findings of tooth luxation and avulsion have been summarized in Table 2. Other important findings for a specific luxation or avulsion are listed below.

**Table 2:** Clinical and radiographic evaluation

Evaluations		Concussion	Subluxation	Extrusive luxation	Intrusive luxation	Lateral luxation	Avulsion
Clinical evaluation	Percussion	Tender	NR	NR	NR	NR	N/A
	Gingival bleeding	−	+/−	+	+	+	+
	Mobility	−	+	+	Immobile	Immobile	N/A
	Dislocation	−	−	+	+	+	+
Radiographic evaluation	X-ray type	Periapical	Periapical	Periapical	1st Periapical 2nd CBCT	1st Periapical 2nd CBCT	Periapical
	Dislocation	−	−	+	+	+	N/A
	Periodontal space	−	−/+	Widen	Reduced/disappear	Widen one side, reduce the other side	N/A

*NR* not recommended (because it causes pain), *N/A* not applicable, *x/x* major/minor (i.e., +/− = positive (major)/negative (minor))

##### Concussion

**Fig. 2:**
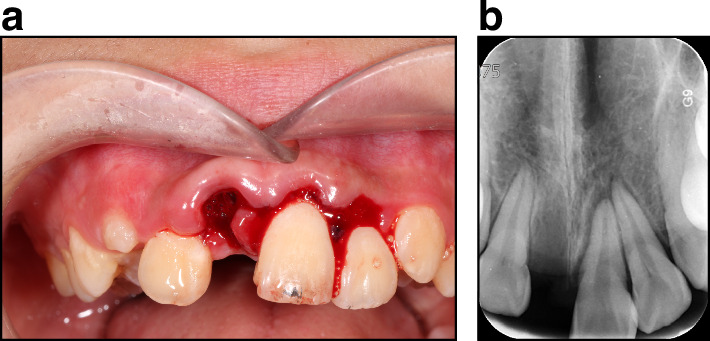
Concussion (Tooth #12, by FDI notation system), a subluxation (Tooth #22), extrusive luxation (Tooth #21), and avulsion (Tooth #11). **a** Clinical exam. **b** Radiograph exam

The tooth may be tender to percussion with minor gingiva bleeding, but mobility is within normal limits. The pulp sensibility testing is usually positive^6^ (Fig. 2).

##### Subluxation

The pulp sensibility testing may be negative, indicating a transient pulp shock. Usually, no radiographic abnormalities are detected. However, in some cases, increased periodontal ligament space may be observed (Fig. 2).

##### Extrusive luxation

The tooth displaced its socket in the incisal/axial direction and appears supper-erupted compared to the original position with increased mobility. The pulp sensibility testing is usually negative. Increased periodontal ligament space in the apical region is usually noted (Fig. 2).

##### Intrusive luxation

Tooth intrusion It is usually associated with the alveolar socket wall fracture or compression.^6^ The pulp sensibility testing is usually negative. The periodontal ligament space is reduced or even absent. The cementoenamel junction dislocates apically than the adjacent non-injured tooth. In severe cases, the injured teeth can be embedded completely sublingually.

##### Lateral luxation

The tooth is displaced in a lateral direction, including palatal/lingual, labial/buccal, or mesial/distal direction, and it is usually associated with the alveolar socket wall or facial cortical bone fracture or compression.^6^ The tooth is usually immobile, but it can be mobile in some cases. If the tooth is stuck, it would give a high metallic (ankylotic) sound by percussion. The pulp sensibility testing is usually negative.

##### Avulsion

Gingival crevice bleeding is present, with gingiva laceration. The alveolar socket is empty.^7^ Sometimes, alveolar socket wall fracture can be observed in radiographic exams (Fig. 2).

**Fig. 3:**
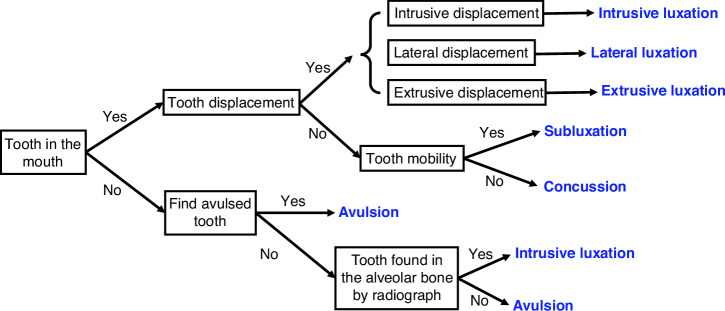
Tooth luxation and avulsion diagnosis decision tree

The diagnosis tree of tooth luxation and avulsion injuries has been summarized in Fig. 3.

## Treatments

### Dental treatments of tooth luxation and avulsion

#### Concussion

No treatment is needed at the first dental visit. For immature primary teeth or very young permanent teeth, the pulp status is usually good.^16^ Therefore, monitoring the pulp condition for six months or up to one year would be adequate to determine if the pulp survives or not. For mature primary teeth, monitor the pulp condition for at least one year. If the pulp becomes necrosis, symptoms, such as gingival pustules, abnormal tooth mobility, abnormal root resorption, and apical periodontitis, would develop. For mature permanent teeth, monitor the pulp condition for at least one year,^6^ or up to two years on the safe side.

#### Subluxation

If the mobility of the tooth is within Grade 1, and the patient feels comfortable with the loose tooth, no treatment is needed.^6^ If the mobility of the tooth is more than Grade 1, or the patient feels uncomfortable with the loose tooth, a passive and flexible splint is recommended to stabilize the tooth for 2 weeks. The risk of pulp necrosis in subluxation is higher than that in concussion. An immature tooth usually has a better outcome than a mature tooth.^43^ Monitor the pulp condition for at least one year or even longer.^6^

#### Extrusive luxation

There is a dislocation in the extrusive luxation. Therefore, the tooth needed to be repositioned and stabilized with a passive and flexible splint for 2 weeks. The risk of pulp necrosis in extrusive luxation is higher than that in concussion and subluxation, especially for a mature tooth with severe dislocation. An immature tooth usually has a better outcome than a mature tooth. Monitor the pulp condition for at least three years or even longer.^6^

#### Intrusive luxation

The treatment of IADT guidelines for an intrusive tooth is summarized in Table 3.^6^ The immature tooth listed in Table 3 is an “ideal” intrusive tooth, with a very young stage of tooth development and the direction of intrusion is exactly apical without any lateral dislocation. However, in a clinic, the situation varies. Make the treatment plan based on the specific situation.

**Table 3:** Treatment plan for intrusive luxation

Tooth maturation	Intrusion severity	Reposition method		
		Spontaneous	Orthodontic	Surgical
Immature	No matter	✓ (4 weeks)	✓ (after 4 weeks)	
Mature	Up to 3 mm	✓ (8 weeks)		
	3–7 mm		✓	✓
	More than 7 mm			✓

#### Lateral luxation

There is a dislocation in the lateral luxation, the tooth needed to be repositioned with a gentle force under local infiltration anesthesia. In the repositioning process, if the tooth is stuck in the alveolar bone, it can be disengaged by a force in the occlusal direction. The tooth should be fixed with a passive and flexible splint for 4 weeks since the alveolar process or alveolar bone fracture is accompanied by lateral luxation. If excessive mobility exists after a 4-week splint, the fixation time can be extended.^7^ An immature tooth usually has a better outcome than a mature tooth, but in general, the dental pulp prognosis is poor. For the immature tooth, especially for very young permanent teeth, revascularization could occur. In this case, the dental pulp is not supposed to be removed at the first dental visit, but the condition of the dental pulp should be monitored in the follow-up visit. If there are signs of necrotic, the dental pulp should be removed. According to the IADT guidelines, for mature teeth, the risk of necrotic dental pulp is relatively high, therefore, the dental pulp should be removed for the prevention of inflammatory external resorption.^6^

#### Avulsion

As mentioned above, the treatment plan for an avulsed tooth varies based on whether the tooth has been replanted before the clinic visit, if not, how long has it been kept dry? It also varies based on whether the tooth is a mature tooth or an immature one. Generally, the treatment plan has been listed in Table 4. For the step-by-step management of an avulsed tooth, please refer to the IADT guidelines.^7^

**Table 4:** The management of an avulsed tooth

Dry time	Apical development		
	Mature tooth	Adolescent tooth	Very young tooth
Put back immediately	PM observation + RCT	PM observation + apexification/apical barrier	PM observation + pulp observation (1st)/endodontic treatment (2nd)
Dry < 60 min	Clean root surface + RCT	Clean root surface + apexification/apical barrier	Clean root surface + pulp observation (1st)/endodontic treatment (2nd)
Dry > 60 min	Clean root surface + RCT	Clean root surface + apexification/apical barrier	Clean root surface + pulp observation (1st)/endodontic treatment (2nd)

*PM* periodontal membrane, *RCT* root canal therapy

### Use of antibiotics

Antibiotics have been widely used in infectious disease control, and the discovery of antibiotics has saved thousands of lives. However, since bacteria can generate resistance to antibiotics, antibiotics should only be prescribed when there is a confirmed infection.^44^ For tooth luxation, since the tooth has not fallen out of the mouth, there is no indication for antibiotics. For tooth avulsion, since the tooth has fallen onto the floor and got contaminated by the soil and bacteria, antibiotics are recommended to control or prevent infection-related inflammation and root resorption.^7^

#### Systemic antibiotics

The use of systemic antibiotics is recommended for tooth avulsion.^7^ Ask patients and caregivers for potential medical history of drug allergy and food allergy. Make sure to calculate children’s weight for antibiotics prescription. Amoxicillin and penicillin are the first antibiotics choice because of their broad-spectrum antibacterial effects and relatively lower risk of side effects.^45^

#### Topical antibiotics

There are two ways of topical antibiotic application. The first one is to apply antibiotics on the root surface before tooth replantation, while the second one is to apply antibiotics in the root canal. No significant improvement in clinical revascularization success rate has been detected in topical antibiotics application.^46^ Theoretically, antibiotics only play a role when there is a confirmed bacterial infection. For the avulsed tooth, if the bacterial infection is mild, such as the tooth failing on relatively clean ground or the tooth has been sent to a dental clinic very quickly in a physiologic media, the effect of antibiotics application would not be that significant.^47^ Dentists should also weigh the pros of minimizing infection by topical antibiotics application and the cons of prolonged tooth replantation by soaking the root in antibiotic solution. For very young avulsed teeth, since the pulp should be preserved, root canal antibiotics application is not practical. For mature avulsed teeth, theoretically, antibiotics can be kept in the root canal to control or reduce root resorption after the replantation.^48^ Further experiments should be conducted to test this hypothesis.

### Tetanus vaccine

Tetanus is a bacterial infection disease, which causes muscle spasms and autonomic nervous system dysfunction. The spasms usually last for several minutes and may cause bone fractures in some severe cases.^49^ Tetanus recovers in several months, but in some cases, it may cause death. It is vaccine-preventable and is rare in developed countries since people have received the vaccine in their childhood and boosters when they grow up.^50^ But on the safe side, a patient with a replanted avulsed tooth is recommended to refer to his physician to check if he needs a tetanus booster. In other luxation situations, since the tooth has not fallen on the ground, there is no need for a tetanus booster.^7^

## Emergency management and consultation on post-trauma care

### Emergency management

**Fig. 4:**
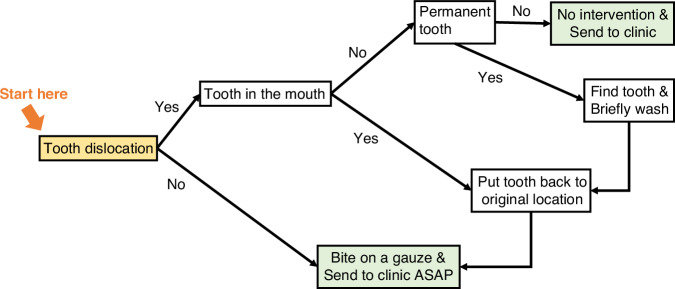
The decision tree for emergency management of tooth luxation and avulsion

When an emergency happens, the caregiver of children or adolescents would be the person who provides first-aid. For many emergencies, timely management by a general practitioner is more important than late management by a medical professional, i.e., the airway, breathing, and circulation management of resuscitation. The goal of emergency management is to relieve the emergency, to minimize side effects, to gain a better prognosis, or at least to gain more time before a medical professional arrives.^51^ In general, traumatic injuries can be divided into penetrating, blunt, decelerating, and thermal injuries.^52^ Blunt and deceleration injuries include general impacts from projectiles, such as falls, TDIs, etc. The goal of emergency management of a traumatic tooth is to preserve the viability of periodontal ligament and dental pulp, in the hope of a better prognosis. In most luxation and avulsion injuries, timely replanting the injured tooth back into the alveolar socket is critical for a better prognosis of the tooth.^6,53^ The emergency treatment has been summarized (Fig. 4).

#### Concussion

No emergency management is needed.

#### Subluxation

Ask the patient to bite on a piece of gauze, a napkin, or a piece of soft clothing to stabilize the tooth and make the patient feel more comfortable before a dental visit.

#### Extrusive luxation

Carefully reposit the extrusive tooth back into its original position. Then, ask the patient to bite on a piece of gauze, a napkin, or a piece of soft clothing to stabilize the tooth.

#### Intrusive luxation

No tooth repositioning is recommended. Ask the patient to bite on a piece of gauze, a napkin, or a piece of soft clothing if the tooth is mobile or an occlusal interference is present.

#### Lateral luxation

Carefully reposit the lateral moved tooth back into its original position if possible. Then, ask the patient to bite on a piece of gauze, a napkin, or a piece of soft clothing to stabilize the tooth.

#### Avulsion

Carefully reposit the avulsed tooth back into its original position if possible. Then, ask the patient to bite on a piece of gauze, a napkin, or a piece of soft clothing to stabilize the tooth.^7,53^ However, if the situation does not allow for repositioning, keep the avulsed tooth in a physiologic media and send the patient to a dental office as soon as possible.^7^ The physiologic media include but are not limited to saline, milk, HBSS (buffer), saliva, propolis solution, oral rehydration salts, rice water, and cling film.^29^

### Post-trauma care

#### Oral hygiene

Meticulous oral hygiene is recommended by the IADT guidelines to maintain the oral health of a dental trauma patient.^2^ Both the patient and the parent should be informed of the oral hygiene details. Especially, for children and adolescents, the application of an antibacterial agent mouthwash for 1–2 weeks is recommended, and for very young children, a cotton swab with an antibacterial agent for 1–2 weeks is recommended.^2^ Like the orthodontic bracket, the flexible splint can obstruct biofilm removal and oral hygiene maintenance.^54^ Therefore, a patient who wears a splint can use an orthodontic toothbrush to clear his tooth, and fluoride varnish should be applied to a patient who wears a prolonged splint.

#### Psychological counseling

Dental and orofacial trauma impacts patients physically and psychologically. One systematic review suggests that trauma has a great impact on a patient’s oral health-related quality of life (OHRQoL), especially, since the prevalence of OHRQoL is 88.4% among adolescents.^55^ The occurrence of dental trauma may impair the emotional balance, social functioning, and well-being of the patient.^56^ Therefore, it is necessary to pay attention to the patient’s psychological changes, such as taciturn or sensitivity. Cooperating with the parents is a simple but effective way to reassure the patient, because parents can provide strong emotional support to the patient. Make both the parents and patient feel the traumatic situation has been properly managed and everything is under control.^57^ Try to positively reinforce the family, such as praising them for having sent the patient to the dental clinic on time before the situation gets worse, and give objective positive signs. Nevertheless, if the dentist feels the psychological problem is out of his control, referring the patient to a psychologist is a wise choice.

#### On-time recalls

On-time recalls ensure proper post-trauma clinical and radiographic observations. If signs or symptoms have been observed, intervention can be applied in time.

### Possible TDI prevention strategies

Epidemiologic studies have revealed that maxillary central incisors are at the highest risk of dental trauma, and falls are the chief reason. Protruded anterior tooth (overjet) and gender significantly contribute to the occurrence of dental trauma.^58^

#### Mouthguard

People who are participating in sports, especially in contact sports and winter sports such as skiing, are at high risk of dental trauma.^59^ It is necessary to inform the sport's participants to wear mouthguards.

#### Orthodontic treatment

The protruded anterior tooth can be corrected by orthodontic treatment, especially for patients who have suffered two or more times on the same protruded anterior tooth.

## Follow-up, prognosis, and management of complications

### Follow-up

The purpose of follow-up is to monitor the status of the injured tooth and whether interventions should be applied. The follow-up time points of the primary tooth and permanent tooth have been listed in Table 5. The timeline of follow-up follows IADT guidelines with some modifications.^2^ We have unified the first follow-up time point of permanent and primary tooth luxation to 2 weeks. The follow-up time point of 8 weeks in primary tooth avulsion has been removed since the healing of gingival laceration has already been estimated in 2 weeks follow-up. If the root cancels therapy of the permanent tooth or pulpectomy of the primary tooth has been done, the risk of pulp infection-related root resorption is low. Therefore, the 8-week follow-up time point of extrusion and lateral luxation has been removed. Moreover, the yearly follow-up has been changed to every other year after one year because the traumatic situation usually becomes stable after one year (Table 5).

**Table 5:** Follow-up time points for tooth luxation and avulsion

Tooth type	TDI type	Time after TDI							
		2 weeks	4 weeks	8 weeks	3 months	6 months	1 years	3 years	5 years and above
Primary tooth	Concussion	*		*		*	*		
	Subluxation	* (S)		*		*	*		
	Extrusion	* PET S		*			*		
	Lateral luxation	*	*PET S	*		*	*		
	Intrusion	*		*		*	*		Up to 6 years
	Avulsion	*							Up to 6 years
Permanent tooth (mature)	Concussion	*R	*R		*R	*R	*R	*R	
	Subluxation	*R (S)			*R	*R	*R	*R	
	Extrusion	*R S	*R RCT		*R	*R	*R	*R	*R
	Lateral luxation	*R	*R RCT S		*R	*R	*R	*R	*R
	Intrusion	*R	*R (S)	*R	*R	*R	*R	*R	*R
	Avulsion	*R RCT S	*R		*R	*R	*R	*R	*R
Permanent tooth (very young)	Concussion	*R					*R		
	Subluxation	*R (S)			*R	*R	*R	*R	
	Extrusion	*R S	*R		*R	*R	*R	*R	*R
	Lateral luxation	*R	*R S		*R	*R	*R	*R	*R
	Intrusion	*R	*R	*R	*R	*R	*R	*R	*R
	Avulsion	*R	*R S	*R	*R	*R	*R	*R	*R

*S* splint removal, *R* radiograph advised even if no clinical signs or symptoms, *PET* pulpectomy, *RCT* root canal therapy

*Clinical review appointment

### Prognosis

#### Concussion and subluxation

Usually good. Monitor the pulp status in follow-up visits. If the pulp becomes necrotic, start the endodontic treatment.

#### Extrusive luxation, intrusive luxation, lateral luxation, and avulsion

Depends on the specific situation. If the pulp is preserved, monitor the pulp status in a follow-up visit. If the pulp becomes necrotic, start the endodontic treatment.

### Treatment of necrotic dental pulp

#### Pulp regeneration and pulp revascularization

The goal of pulp regeneration, also known as regenerative endodontics, is to regenerate the pulp–dentinal complex. Basic research and preclinical research have successfully used dental pulp stem cells (DPSCs) and dental mesenchymal stem cells to regenerate the dental pulp. But at this moment, in clinical practice, it is pulp revascularisation rather than pulp regeneration. The pulp revascularisation restores the pulp chamber with blood vessels, connective tissues, and some pulp cells but the pulp–dentinal complex has not been regenerated.^13^ Pulp revascularization is recommended for necrotic very young permanent teeth. Detailed pulp revascularisation protocol can be found in the European Society of Endodontology position statement.^60^

#### Apical barrier

The apical barrier is recommended for very young permanent teeth and adolescent teeth. It uses biocompatible materials to seal the open apical foramen, therefore, the filling materials will be confined to the root canal.^61^

#### Apexification

Apexification is recommended for most types of immature teeth, and if other treatments, such as pulp revascularisation and apical barrier, fail. Unlike apical battier, the apexification requires frequent recalls to change the calcium hydroxide sealed in the root canal.^62^ If frequent recalls are impracticable, the patient is recommended for an apical barrier.

#### Root canal therapy

**Fig. 5:**
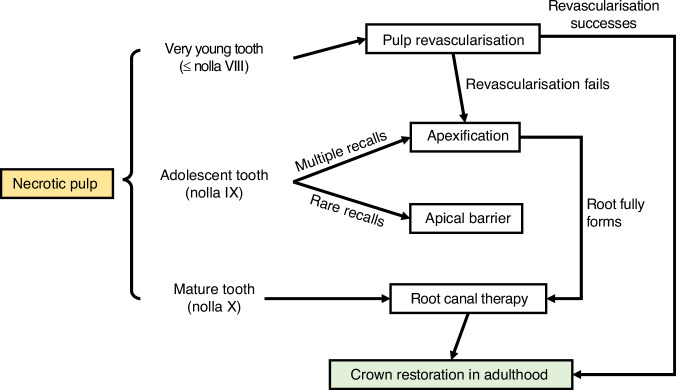
The decision tree for necrotic pulp management

Root canal therapy is recommended for mature teeth. The crown of the traumatic tooth should be restored in the patient’s adulthood (Fig. 5).

### Treatment of ankylosed tooth

#### Observation

If the ankylosis happens to an adult, whose craniofacial development is finished, the tooth might not get submerged and could still maintain the normal occlusion. In this case, no intervention is needed.

#### Crown restoration

If the ankylosis happens to a child or an adolescent, whose craniofacial development is not finished, the tooth might get submerged. In this case, a crown restoration can be used to extend the clinical crown length.

#### Tooth decoration and dental implant

If the root is severely resorbed, with the disappearance of periodontal ligament space, and the cementum fused with the alveolar bone, tooth decoration should be conducted. In tooth decoronation, the crown portion of the tooth is removed and the root canal is filled with blood drained from the periapical connective tissue. Over a certain time, the root structure disappears, but the alveolar width has been maintained. This is a conservative transitional method of bone width and height preservation. When the patient becomes an adult, the dental implant can be conducted.^63^

#### Orthodontic treatment

Please refer to the “Considerations for dislocated dental injuries in orthodontic treatment planning” section of this consensus.

## Considerations for dislocated dental injuries in orthodontic treatment planning

### Tooth root movement

#### Assessment of the health status of traumatized teeth

A detailed evaluation of traumatized teeth is required, encompassing the health of the dental pulp, the integrity of the root structure, and the healing process of the tooth and surrounding tissues.^64^ This includes assessing the extent of root resorption that has occurred, the stability of the tooth, and the condition of the periodontal tissues.^65^

#### Selecting the appropriate timing for treatment

It is crucial to determine the most suitable start time for treatment to ensure ample recovery of the teeth and surrounding tissues. For the orthodontic movement of traumatized teeth, it is recommended to wait for a period post-trauma (such as 3 months to 1 year) to allow for the natural healing process to take place.^65^

#### Considering the limitations of tooth movement

Teeth that have been traumatized or dislocated may respond differently to orthodontic forces compared to undamaged teeth. In such cases, tooth movement can be more complex, requiring precise control of forces and movement strategies to avoid further root resorption or other complications due to excessive force.^65–67^

#### Utilizing light forces and progressive orthodontic techniques

**Fig. 6:**
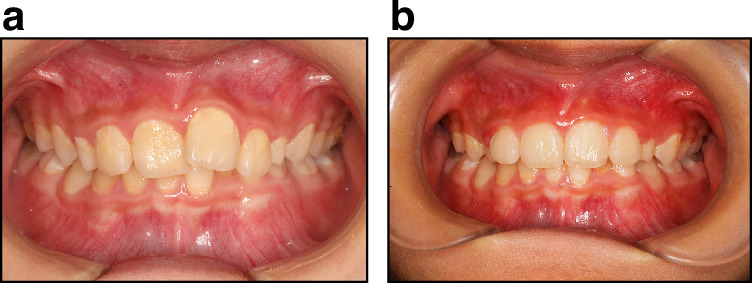
2 × 4 Light force alignment for a half-year traumatic tooth. **a** Before orthodontic treatment. **b** After orthodontic treatment

To minimize the adverse effects of tooth movement on damaged teeth, it is advisable to use light and gradual forces, along with more refined orthodontic methods. Light-force orthodontics can reduce stress on the teeth and periodontal tissues, lowering the risk of further damage (Fig. 6).^65,66^

#### Regular monitoring and treatment plan adjustments

Throughout the orthodontic treatment process, regular monitoring of tooth movement and periodontal health is essential. This may require periodic clinical examinations and radiographic assessments to ensure that tooth movement is proceeding as planned and to adjust the treatment plan when necessary.^66,67^

#### Patient communication and education

Communicating the treatment plan, potential risks, and expected outcomes to patients and their parents is of great importance. Patient understanding and cooperation are crucial for the success of the treatment.^68,69^

### Root resorption

#### Mechanisms of root resorption

Orthodontically induced external apical root resorption (EARR) is a common and detrimental side effect driven by inflammation during orthodontic movement. This type of root resorption can lead to irreversible loss of tooth structure.^70^ Studies indicate that 90% of teeth undergoing orthodontic treatment experience EARR, with over 80% of patients showing more than 1 mm of root resorption post-treatment, and one-third exceeding 3 mm.^71^

#### Timing of orthodontic treatment

For dislocated teeth requiring both endodontic and orthodontic treatments, priority should be given to endodontic treatment as it lays the foundation for subsequent orthodontic procedures.^72^ The optimal timing for orthodontic tooth movement depends on the healing of apical lesions post-endodontic treatment and the extent of trauma to the tooth. Comprehensive clinical assessment is crucial in selecting the most appropriate treatment method for optimal outcomes.^71^

#### The interval between orthodontic and endodontic treatments

For mildly traumatized teeth, a waiting period of 3–6 months may be necessary to allow the normalization of periodontal tissues and structures before initiating orthodontic movement. For moderately traumatized teeth, a minimum waiting period of 1 year is recommended until the apex radiograph or tomography shows normal results. In severe trauma cases, such as root fractures, the waiting period should be extended to 2 years or more. Studies suggest that starting orthodontic treatment 4–5 months after mild to moderate trauma does not increase the risk of root resorption.^71^

#### Teeth post-endodontic treatment and orthodontic treatment

Teeth post-endodontic treatment (RFT) exhibit relatively lower EARR compared to vital pulp teeth after orthodontic treatment. Therefore, initiating orthodontic movement post-endodontic treatment is considered a relatively safe approach. When both treatments are necessary, endodontic treatment should precede. The timing of orthodontic treatment should be determined based on the healing of the apical lesion and the severity of the trauma, and it is advisable to commence when the apical X-ray appears normal.^71^

### Dental ankylosis

#### Diagnosis and assessment

Initially, an accurate diagnosis and assessment of the extent and range of dental ankylosis are required. This involves clinical examination (such as the metallic sound heard on percussion) and radiographic examination (such as X-rays, showing fusion between the tooth and the alveolar bone).^73^ This assessment helps to determine the mobility of the tooth and the feasibility of orthodontic treatment.^66^

#### Considering the limitations of tooth movement

In cases of dental ankylosis, orthodontic movement of the tooth will be limited. The tooth may be difficult to move using traditional orthodontic methods, as the fusion of the ankylosed tooth with the alveolar bone hinders the normal remodeling process of the periodontal bone.^66^

#### Exploring alternative treatment methods

In some cases, alternative treatment methods, such as surgical procedures (like surgical luxation or distraction osteogenesis) to break the fusion between the tooth and the alveolar bone, may need to be considered to allow tooth movement. These methods can be more complex and require precise planning and execution.^66^

#### Considerations of esthetics and function

In designing orthodontic treatment plans, special attention should be given to esthetic and functional requirements. For ankylosed teeth, it may be necessary to consider the movement of other teeth to improve the overall dental alignment and occlusion, while maintaining or enhancing esthetics.^74,75^

#### Long-term monitoring and management

Orthodontic treatment of ankylosed teeth requires long-term monitoring and management. Periodic radiographic assessments and clinical examinations may be needed during treatment to monitor the progress of tooth movement and the health of the teeth.^66^

#### Instructions for patients and parents

For children and adolescent patients, it is crucial to explain the nature of dental ankylosis, the challenges of treatment, and the expected outcomes to patients and their parents. Ensuring that they understand the treatment process, potential complexities, and possible outcomes helps establish appropriate expectations and a collaborative relationship.^66^

### Other factors

In orthodontic treatment planning for teeth re-implanted after dislocation, besides considering root movement, root resorption, and dental ankylosis, the following key aspects should be meticulously considered:

#### Biological recovery of the tooth

Re-implanted teeth may require a period of biological recovery and stabilization. Before initiating orthodontic treatment, it is crucial to ensure that the periodontal environment of the tooth has sufficiently healed and the health of the pulp has been confirmed.^76^

#### Assessment of tooth stability

The stability of reimplanted teeth needs to be evaluated. If the tooth is not adequately stable in its socket, a longer period may be required for stabilization, or auxiliary fixation methods might be considered to provide additional support.^76^

#### Sensitivity testing of the tooth

Reimplanted teeth may be more sensitive to temperature or pressure. This increased sensitivity needs to be considered in the treatment plan to avoid unnecessary discomfort or pain during orthodontic treatment.^76^

#### Consideration of tooth position and adjacent teeth

The position of reimplanted teeth and their occlusal relationship with adjacent teeth might differ. When designing orthodontic plans, the alignment and positional relationships of the entire dental arch, and how to effectively incorporate the reimplanted tooth, should be considered.^65^

#### Consideration of tooth position and adjacent teeth

The position of reimplanted teeth and their occlusal relationship with adjacent teeth might differ. When designing orthodontic plans, the alignment and positional relationships of the entire dental arch, and how to effectively incorporate the reimplanted tooth, should be considered.^77^

#### Regular monitoring and adjustments

Throughout the orthodontic treatment process, regular monitoring of the tooth’s response and progress is needed, with timely adjustments to the treatment plan based on the tooth’s movement and reactions.^77^

Orthodontic treatment of teeth with dislocation injuries involves not only considerations of physical tooth movement but also a comprehensive assessment of the tooth’s biological response, the health of surrounding tissues, and potential risks. These considerations together form the basis for effective, safe, and successful orthodontic treatment of patients.

## Conclusion and expectation

The incidence of tooth luxation and avulsion is still high worldwide, and about one-third of the world's population has suffered TDIs.^2^ The traumatic injuries cause pain and uncomfortable, bring economic burdens, compromise quality of life, and generate trauma-related dental anxiety.^2^ Therefore, timely and proper clinical management would benefit the prognosis of the traumatic tooth, and this is the secondary prevention, the goal of which is to minimize complications and promote healing. On the other hand, preventing the occurrence of TDIs, known as primary prevention, by wearing a mouthguard and other protective equipment would be another goal of TDI prevention. Patients and physicians can also refer to the ToothSOS App, which was developed by the IADT, for emergency management guidelines. Future studies could focus on pulp regeneration techniques, methods of ankylosis prevention, orthodontic treatment of a traumatic tooth, propaganda of public awareness of TDI prevention, and first-aid procedures.
